# Quantitative real-time PCR (qPCR) for *Eimeria tenella* replication — Implications for experimental refinement and animal welfare

**DOI:** 10.1016/j.parint.2015.06.010

**Published:** 2015-10

**Authors:** Matthew J. Nolan, Fiona M. Tomley, Pete Kaiser, Damer P. Blake

**Affiliations:** a Department of Pathology and Pathogen Biology, Royal Veterinary College, University of London, Hatfield AL9 7TA, United Kingdom; b The Roslin Institute, University of Edinburgh, Easter Bush, Midlothian EH25 9RG, Scotland, United Kingdom

**Keywords:** Avian coccidiosis, Chicken, RAPD-SCAR marker (Tn-E03-1161), Cytoplasmic beta-actin gene (*actb*)

## Abstract

The *Eimeria* species are highly pathogenic parasites of chickens. Research aimed at reducing their impact is hindered by a lack of non-subjective, quantitative, tools to measure parasite replication in the host. The time-consuming, and often time-sensitive, nature of existing approaches precludes their use in large-scale genetic, epidemiological, and evolutionary analyses. We have used quantitative real-time PCR (qPCR) to accurately quantify *Eimeria tenella* in chicken tissue and shown this to be more efficient and sensitive than traditional methodologies. We tested four chicken-specific reference qPCR assays and found beta-actin (*actb*) to be optimal for sample normalisation. In an experimental setting, chickens were inoculated with 500, 1500, or 4500 *E. tenella* oocysts and parasite replication and the impact of infection measured by i) qPCR analysis of DNA extracted from caecal tissues collected at five and eight days post-infection (dpi), ii) faecal oocyst counts (FOCs) on samples taken from six to eight dpi, and iii) lesion scoring on caeca collected post-mortem at five and eight dpi. Quantitative real-time PCR test results indicated a significant dose-dependent increase in parasite numbers among study groups for samples collected five dpi (i.e., prior to gametogony) (*R*^2^ = 0.994) (*p* < 0.002) but not in those from day eight (after most oocyst shedding) (*R*^2^ = 0.006) (*p* > 0.379). A strong dose-dependent increase in parasite replication and severity of infection was also revealed by FOC (*R*^2^ = 0.997) and lesion scoring. Importantly, qPCR offers substantial improvements for animal welfare via improved statistical power and reduced group sizes in experimental studies. The described qPCR method overcomes subjective limitations of coproscopic quantification, allows reproducible medium- to high-throughput examination of tissues, faeces, and oocysts, and is a valuable tool for determining the impact of *Eimeria* infections in both experimental and field settings.

## Highlights

• Quantitative PCR indicates significant dose-dependent increases in parasites.
• More sensitive measure of parasite replication than faecal oocyst count/lesion score
• Significant implications for animal welfare and experiment refinement (3R's)
• Support studies focusing on the genetic basis of resistance/susceptibility
• Major implications for investigations of key aspects of *Eimeria* biology and control

## Graphical abstract

**Figure:**
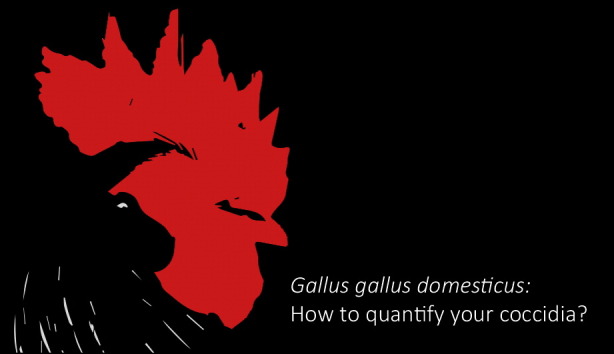

## 1 Introduction

Chicken coccidiosis is caused by seven strictly host-specific species of *Eimeria* (Apicomplexa, Eimeriidae; [1]). Disease severity depends on variables such as magnitude of dose, parasite and host genotype, and host immune status. Symptoms of coccidiosis may include oedema of the submucosa, stimulation of glandular tissues, thickening of the intestinal walls, villus atrophy, and in severe cases complete villus destruction resulting in extensive haemorrhage and death [2,3]. Current control of *Eimeria* relies primarily on the administration of routine chemoprophylaxis and, to a lesser extent, vaccination with live wild-type or attenuated parasites. However, the development of multi-drug resistant strains [4–6], and the relative cost and limited production capacity for live vaccines, has undermined the portfolio of effective treatment options [7]. In addition to chemical management, specific diagnosis and an understanding of parasite replication is critical for control and surveillance of coccidiosis [8]. Presently, quantification of *Eimeria* relies on faecal or litter oocyst counts (FOCs) [9–13] or protracted microscopic examination of stained tissue sections (intracellular parasite stages), while species identification commonly requires assessment of oocyst morphology, or lesions and site of infection during post-mortem [2]. These techniques are robust when carried out by skilled individuals although they can be subjective, time consuming, and difficult to scale up for medium to high-throughput applications [14–16].

The persistent threat of coccidiosis highlights an urgent need for quantitative tools that can rapidly and accurately determine parasite numbers in both field and experimental investigations. Innovative molecular approaches have included the application of polymerase chain reaction (PCR) assays that are specific, objective, and efficient but are qualitative, and rely on visual interpretation of stained agarose gels [17]. More recently genus- and species-specific quantitative real-time PCR (qPCR) has been applied [18,19], which is a high-throughput fluorescence-based method of enzymatic amplification in a ‘closed-tube’ format that can detect and measure minute quantities of DNA [20–22] and calculate genome or transcript copy number [23]. The utility of this technique to study *Eimeria* and other apicomplexans (e.g., *Cryptosporidium* [24], *Isospora* [25], and *Toxoplasma* and *Cyclospora* [26]), is due to its sensitivity, specificity, and capacity to quantify different parasites in excised tissue, faecal samples, and purified parasite suspensions [27]. Here, we critically evaluate the reliability and suitability of qPCR for large-scale quantitative investigations of *Eimeria tenella* in experimentally infected chickens. We directly compare our test results to the traditional McMaster oocyst counting, and lesion-scoring techniques, as an entrée to using qPCR for novel large-scale genetic, epidemiological and evolutionary studies.

## 2 Materials and methods

### 2.1 Chicken management

Forty, two-week-old specific pathogen-free (SPF) Lohmann white chickens (*Gallus gallus domesticus*) were housed with environmental enrichment in coccidia-free conditions and allowed to acclimatise for seven days. For the duration of this study birds were observed twice per day for signs of illness and/or welfare impairment and were housed, handled, and treated following Home Office regulations under the Animals (Scientific Procedures) Act 1986 (ASPA), and the guidelines laid down by the Royal Veterinary College ethics committee.

### 2.2 Parasite preparation and inoculation

Sporulated *E. tenella* oocysts of the Wisconsin (Wis) reference strain [28] were produced and maintained as described previously to generate inocula of 1000, 3000, and 9000 sporulated oocysts per ml [10].

At experimental day 0, three-week old chickens were randomly assigned to four groups. Four birds (group 1) were maintained as study controls and were not inoculated, while the remaining 36 chickens were equally divided among three groups and inoculated via oral gavage with 0.5 ml of one inoculum receiving a single dose of 500 (group 2), 1500 (group 3), or 4500 (group 4) sporulated oocysts.

### 2.3 DNA standard dilution series

Tenfold DNA standard dilution series representing *E. tenella* or chicken total genomic DNA (gDNA) were prepared as described previously [29]. In brief, the concentration of each gDNA sample was determined by NanoDrop spectroscopy in a ND-1000 spectrophotometer (Thermo Scientific, Wilmington, USA) and by comparison with known standards resolved via agarose gel electrophoresis in triplicate [30]. Based upon predicted genomes sizes of 51.8 Mbp (*E. tenella*) [31] and 1.2 Gbp (*G. domesticus*) [32,33] genome copy number was determined per microliter (using Avogadro's number 6.022 × 10^23^ molecules/mol and the average weight of a base pair of 660 g/mol), to generate the standard dilution series using glycogen (Thermo Scientific) as a carrier (final concentration of 33 μg/ml; [34]). Dilution series ranged from 10^4^–10^0^ genome copies per ml.

### 2.4 Primer selection, PCR amplification and sequencing of host qPCR targets to determine specificity

Genomic DNA was purified from *E. tenella* oocysts and uninfected chicken intestinal tissue as described previously [19] and subjected to PCR amplification and sequencing to determine primer specificity. For quantification of *E. tenella* genome copy number we used the published primers Ete_qPCRf (forward: 5′-TCGTCTTTGGCTGGCTATTC-3′) and Ete_qPCRr (reverse: 5′-CAGAGAGTCGCCGTCACAGT-3′) [19], targeting the *E. tenella* RAPD-SCAR marker Tn-E03-1161 [35]. Four other primer pairs (see Table 1), targeting portions of the chicken cytoplasmic beta-actin (*actb*), beta-2 microglobulin (*β_2_m*), glyceraldehyde 3-phosphate dehydrogenase (*gapdh*), and tata-binding protein (*tbp*) genes, were evaluated for their suitability as reference sequences for the purposes of normalisation.

PCR amplification of 121 nucleotides of Tn-E03-1161 was achieved with an established protocol [19]. A portion of each *actb*, *β_2_m*, *gapdh*, and *tbp* locus were amplified in a volume of 50 μl containing 20 mM Tris–HCl (pH 8.4) and 50 mM KCl (10 × PCR buffer, − Mg), 3.0 mM of MgCl_2_, 0.2 mM of each deoxynucleotide triphosphate, 50 pmol of each primer, 1.25 U of *Taq* DNA polymerase, recombinant (Invitrogen™, Life Technologies, Carlsbad, USA), and 2.0 μl of gDNA utilising the cycling protocol 95 °C/5 m (initial denaturation), followed by 35 cycles of 95 °C/30 s (denaturation), 60 °C/30 s (annealing), 72 °C/30 s (extension), followed by a final extension of 72 °C/10 m. Visualisation of PCR amplicons was achieved on 1.5% w/v agarose in TBE (tris, boric acid, ethylenediaminetetraacetic acid [EDTA] buffer) gel stained with SafeView Nucleic Acid Stain (Novel Biological Solutions, Huntingdon, UK). In brief, 5 μl of each amplicon was mixed with 1 μl of 6 × DNA loading Dye (Thermo Scientific) and then subjected to electrophoresis at 50 V for 1 h using TBE buffer (0.89 M tris base, 0.89 M boric acid, 0.5 M EDTA; Sigma Aldrich, USA). A GeneRuler Low Range DNA Ladder (Thermo Scientific) was included on each gel for size comparison purposes. All PCR amplicons were purified using a QIAquick® PCR Purification Kit (Qiagen, Hilden, Germany), according to the manufacturer's instructions. Purified amplicons were then subjected to cycle sequencing reactions using ABI Ready Reaction Mix (BigDye® Terminator v3.1 chemistry, Applied Biosystems, USA) and the same primers employed for PCR (separately), followed by direct automated sequencing at GATC Biotech, Cologne, Germany. Sequence quality was verified by comparison with corresponding electropherograms and consensus sequences were constructed using the software package CLC Main Workbench v.6.9.1 (CLC bio, Aarhus, Denmark). Sequence similarity was ascertained by Basic Local Alignment Search Tool analyses (BLAST®: http://blast.ncbi.nlm.nih.gov/Blast.cgi).

### 2.5 Quantification of in vivo *E. tenella* replication

#### 2.5.1 Quantitative real-time PCR (qPCR)

Six birds from each of study groups 2–4, representing half of each inoculated group, were euthanised five days post infection (dpi; 120 h pi) and the remaining 22 birds (the other half of each study group plus the four study controls) were euthanised eight dpi (192 h pi) according to the guidelines of the Home Office regulations under A(SP)A. Immediately upon death the viscera were exposed, the caeca separated from the large intestine, the caecal contents removed, and the complete caecal pair transferred to a 30 ml polypropylene tube containing 5–10 volumes of RNA*later*® (Life Technologies; Carlsbad, CA, USA) at room temperature (RT), as per the manufacturer's instructions. Samples were stored for a period of seven days at 4 °C before the RNA*later*® was decanted and samples stored at − 20 °C.

Total gDNA was isolated from each caecal pair using a DNeasy® Blood and Tissue kit (Qiagen). In brief, caeca were weighed and suspended in an equal w/v of Qiagen tissue lysis buffer. Complete caeca were then homogenised employing a TissueRuptor (Qiagen) and the equivalent of ≤ 25 mg of the homogenate added to a sterile 1.5 ml microcentrifuge tube. Genomic DNA was then extracted as per the manufacturer's instructions for Purification of Total DNA from Animal Tissues. Total gDNA was stored at − 20 °C, until further investigation.

Quantitative real-time PCR was performed using a CFX96 Touch® Real-Time PCR Detection System (Bio-Rad Laboratories, Hercules, California, USA), as described by the manufacturer. Briefly, each sample was amplified in triplicate in a 20 μl volume containing 1 μl of total gDNA, 300 nM of each primer, 10 μl of SsoFast™ EvaGreen® Supermix (Bio-Rad Laboratories), and 8.8 μl of DNase/RNase free water (Gibco™, Life Technologies) with qPCR cycling conditions that consisted of 95 °C/2 m (enzyme activation/initial denaturation), followed by 40 cycles of 95 °C/15 s (denaturation), 60 °C/30 s (annealing/extension), followed by melt analysis of 65 °C–95 °C at increments of 0.5 °C/0.5 s. Each qPCR assay included the relevant gDNA dilution series (standards) and no template controls (NTC), and was conducted employing white hard-shell® 96-well PCR plates (Bio-Rad Laboratories) sealed with Thermo Scientific adhesive sealing sheets.

#### 2.5.2 Collection of faecal material and McMaster oocyst counts

To quantify *E. tenella* replication by FOC we collected total faecal material from six to eight dpi separately from each of the 22 birds that remained after the five dpi sampling to coincide with the period of greatest oocyst excretion [36,37]. Total FOC per bird was determined as described by Shirley [38].

#### 2.5.3 Lesion scoring

Caecal lesion scores were determined prior to tissue preservation from birds culled five and eight dpi employing the method described by Johnson and Reid [39].

### 2.6 Statistics

The copy number of each qPCR target (for each test sample) was calculated based on the slope and intercept generated by the corresponding reference dilution series using qPCR software CFC manager v.3.1 (Bio-Rad Laboratories). Predicted parasite genome copy number in tissue samples was normalised by comparison to the estimated host genome copy number. Samples collected five dpi were analysed independently of those collected eight dpi. The normalised number of parasite genomes per host genome (in one μl) was employed to infer parasite copy number per milligramme of host tissue. Quantification cycle data (Cq) resulting from triplicate qPCR amplification of each test sample, standard, and NTC was averaged and the standard deviation (SD) and relative standard deviation (% RSD) recorded. The efficiency (*E*) of each qPCR assay was determined employing the formula (Eq. (1)) [40]:

(1) $$\mathrm{Efficiency}\;\mathrm{of}\;\mathrm{qPCR}\;\left(E\right)=\left(10^{\;\frac{-1}{\mathrm{slope}}}-1\right)*100\text{.}$$

The arithmetic mean, SD, and % RSD of Cq values for each biological replicate/study group were determined using the programme Excel (Microsoft Corporation, Redmond, Washington, USA). Faecal oocyst count figures were normalised by Log_10_ transformation prior to statistical analysis. Statistical analyses were conducted using the software package IBM SPSS Statistics 22 (IMB, New York, USA) and included one-way ANOVA and the a posteriori Bonferroni and Tukey's tests. Statistical significance of categorical lesion score data was assessed using the Kruskal–Wallis test and the a posteriori Dunn's test. Differences were considered significant with a *p*-value of < 0.05. Power calculations to identify the minimum sample size (number of birds per study group) required to obtain significant FOC were done at the Statistical Solutions LLC website (http://www.statisticalsolutions.net/pss_calc.php) where mu(0) was the average of all of the samples (not including the no parasite control), mu(1) was the average of one group (any), and the default values of 0.05 and 0.8 were employed for ‘alpha’ and ‘power’ (respectively).

In assessing which of four genomic loci were most suitable to serve as reference sequences for the purposes of normalisation we examined assay specificity, repeatability (i.e., short-term precision; see Bustin et al. [23]), and efficiency on three separate occasions. Subsequently, we endeavoured to obtain a standard curve (log [DNA copy number] vs. Cq) with a slope of − 3.322, which would theoretically yield an amplification factor of 2.0 and an assay efficiency of 100%.

## 3 Results

### 3.1 Identification of an optimal chicken genomic DNA reference sequence

Four primer pairs targeting portions of separate protein-encoding loci, which could serve as reference sequences for the purposes of data normalisation, were evaluated for their specificity during PCR by a combined PCR/sequencing/comparative analyses-based approach and by qPCR via melting curve analysis. Visualisation of PCR amplicons by agarose gel electrophoresis indicated that cyclic amplification of each locus had generated a single product that ranged in size from 98–152 base pairs, as expected (see Table 1). Subsequent sequencing and appraisal of resultant genetic data indicated that each amplicon generated in this study was unique. Comparison of these sequences with information available in public databases (i.e., GenBank) revealed that each was 100% identical to the corresponding sequence representing the chicken *actb* (represented by the GenBank accession number X00182; [41]), *β_2_m* (Z48922; [42]), *gapdh* (M11213; [43]), and *tbp* (NM_205103; [44]) genes. To provide further evidence of specificity for each assay, melting curve analysis of amplicons following qPCR indicated that the mean melting temperature of each locus, across 36 reactions (including triplicates), was 83.03 ± 0.1 for *actb*, 82.79 ± 0.3 for *β_2_m*, 87.62 ± 0.4 for *gapdh*, and 83.74 ± 2.0 for *tbp*.

**Fig. 1:**
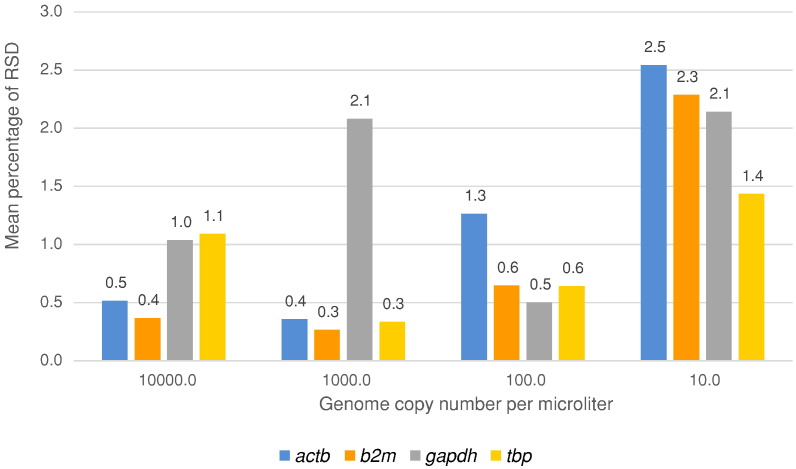
The short-term precision, or intra-assay variability, of qPCR assays assessing four independent unlinked loci as reference genes for the purposes of data normalisation. Figures expressed as the mean relative standard deviation (% RSD) for Cq variance evaluated in three separate assays over a two-week period.

Quantitative real-time PCR assay repeatability and efficiency were evaluated in three separate assays over a two-week period following as many freeze/thaw events. We amplified a single set of linear genomic DNA dilutions over six, then five, orders of magnitude with each sample amplified in triplicate. The short-term precision, or intra-assay variability, was determined by analysis of the mean % RSD for Cq variance (see Fig. 1). Overall, assay precision was comparable for the four loci with % RSD values ranging from 0.4–2.5 for *actb*, 0.3–2.3 for *β_2_m*, 0.5–2.1 for *gapdh*, and 0.3–1.4 for *tbp*. Precision generally decreased with a reduction in genome copy number; this trend appeared stronger (i.e., a greater increase in % RSD with each further dilution of gDNA) for assays amplifying *actb* and *β_2_m* compared to those for *gapdh* and *tbp* (see Fig. 1). Differences in precision among the four loci at each dilution were not significant (*p* > 0.185).

In assessing qPCR efficiency (*E*), analysis of each assay indicated that all reactions resulted in linearly positive correlations with mean coefficients of determination (*R^2^*) of 0.985 ± 0.009 (*actb*), 0.983 ± 0.009 (*β_2_m*), 0.975 ± 0.009 (*gapdh*), and 0.988 ± 0.006 (*tbp*). The mean slope, amplification factor, and efficiency of assays amplifying three of four loci were comparable; amplification of *actb*, *β_2_m*, and *tbp* generated slopes of (ca.) − 3.0 ± 0.2, amplification factors of 2.1 ± 0.1, and efficiencies of 113% ± 13 (see Table 2). In contrast, assays amplifying *gapdh* produced results of − 2.400 ± 0.427 (slope), 2.685 ± 0.533 (amplification factor), and 170.529% ± 54.955 (efficiency).

### 3.2 Comparison of McMaster oocyst counts and lesion scores with quantitative real-time PCR as a measure of parasite replication

McMaster oocyst counts were obtained from total faecal samples collected daily and pooled between six and eight dpi. The arithmetic mean number of oocysts excreted per chicken in each study group increased as initial oocyst dose increased (Table 3), while oocysts were not detected during FOCs on faeces collected from control-group birds (group 1). Oocyst output among the three differently inoculated groups exhibited a strong dose-dependent linear positive correlation (*R^2^* = 0.997; Table 3), although the differences were not statistically significant (*p* > 0.098). Mean lesion scores were also found to increase as oocyst dose increased at both five (groups 2 and 3 significantly different from group 4, *p* = 0.002) and eight dpi (group 2 compared with groups 3 and 4, *p* = 0.001), although not all differences were significant (Table 3). Chickens in the control group did not have lesions.

The trend of increased parasite number versus initial inoculum dose was reflected in the number of intracellular parasite genomes detected five dpi by qPCR with a strongly linear positive correlation (*R^2^* = 0.994; Table 3). A posteriori tests indicated that differences among the means across all groups were significant (*p* < 0.002) (Table 3). Quantitative real-time PCR data generated from samples collected eight dpi did not demonstrate a relationship between residual intracellular parasite genome numbers and initial dose, reflected by the absence of a clear correlation (*R^2^* = 0.006) and no statistically significant differences (*p* > 0.379).

The precision of the qPCR assays, measured as the standard deviation of triplicate Cq values for each sample, was high with variation ranging from 0.017–0.167. This resulted in SD in copy number (prior to normalisation) of as little as 23.8 to 3453.5 genome copies. No amplification was observed for any of the NTC samples, on any occasion. These findings demonstrate the specificity of the PCR conditions employed to amplify the *E. tenella* RAPD-SCAR marker Tn-E03-1161.

## 4 Discussion

This study represents the first validation of an objective, highly sensitive, and efficient published quantitative real-time PCR technique to expedite the process of determining *Eimeria* parasite replication in tissue for both small and large-scale investigations in laboratory and field-place settings. Previous reports using qPCR methods have focused on developing assays for the specific identification of multiple chicken-infecting *Eimeria* species [19,45–48], or for quantification of a single species (i.e., *Eimeria acervulina* [18,27] and *Eimeria maxima* [29]). Here, we directly compared qPCR of tissue samples with FOC and lesion scores in experimentally infected chickens to demonstrate a robust correlation between oocyst dose, qPCR test results, and FOC.

We detected *E. tenella* genomic DNA in all 36 experimentally infected chickens. Quantitative real-time PCR test results from tissues collected five dpi indicated a dose-dependent relationship between the size of inoculum and intracellular parasite genome copy number. By eight dpi, after completion of most oocyst shedding, this relationship was no longer apparent (*R*^2^ = 0.006). FOC and lesion scores also showed strong relationships between inoculum dose and oocyst outputs or lesion scores; however, there was an overall lack of statistically significant differences between groups in FOC while differences in lesion scores were significant between two, but not all doses (Table 3). These findings are of major importance. The low intra-group variation defined by qPCR at five dpi compared to traditional measures of parasite replication, such as FOC or lesion scoring, offer opportunities to reduce experimental group sizes without compromising statistical quality, a reduction in line with the National Centre for the Replacement, Refinement and Reduction of Animals in Research (NC3Rs; http://www.nc3rs.org.uk/) principles. Statistical significance for *E. tenella* commonly requires at least eight replicate birds per experimental group when using measures such as FOC [49]. Here, power calculations using the standard deviation associated with the within-group FOC variation indicated that we would have required group sizes in excess of 20 to detect significant differences in oocyst excretion associated with dose size, compared to just six for qPCR when measured at five dpi.

Biological and technical factors can influence gDNA-based qPCR results and must be considered carefully before this tool can be used widely as a realistic alternative to FOC. In previous qPCR studies with *Eimeria* carried out by Morgan et al. [48] and Raj et al. [45], gDNA was extracted directly from faeces employing (singly or in combination) a QIAamp® DNA Stool Mini Kit (Qiagen), DNeasy® Tissue Kit (Qiagen), and/or a standard cetyl trimethylammonium bromide (CTAB) [50] extraction protocol. These methods are at least partially ineffective at removing faecal components inhibitory to PCR, as demonstrated using an internal positive control (IPC) qPCR in the latter study. Using tissue samples in place of caecal contents or faeces can reduce the risk of inhibition and could be confirmed by inclusion of an IPC assay [45]. Quantification of *Eimeria* genome numbers in tissue, rather than faecal or litter samples, offers the additional benefit of removing sporulation as a variable. Sporogony usually occurs between 24 and 72 h after oocyst excretion, but can be completed in under 24 h under optimal conditions of temperature and moisture [51], introducing at most a four-fold potential for error as the diploid unsporulated oocyst differentiates to eight haploid sporozoites [19]. Efforts to account for such variation have included calculation of a sporulation factor [48] or sample refrigeration to minimise sporulation [45]. Moreover, qPCR using species-specific assays (e.g., [29]) can be of value when assessing the impact of co-infection by more than one *Eimeria* species. The influence of other biological variables, such as the crowding effect whereby parasite fecundity is reduced once a ‘crowding threshold’ has been reached [11,52], are likely to exert equal effects on both qPCR and FOC quantification of faecal oocyst load.

Despite these technical benefits, when quantifying *Eimeria* numbers in tissue samples, it is critical that the timing of infection is known as qPCR targeted at gDNA will not differentiate between a high level of infection early in the parasite's life cycle and a lower level of infection later in the life cycle. Thus, qPCR quantification of *Eimerian* parasites in tissue samples should be considered an excellent replacement for FOC under controlled experimental conditions or in unusual field situations when the time of infection is known. Of particular value will be the reductions in animal usage achieved through the use of smaller experimental group sizes and a substantial reduction in investigator time in large-scale experimental and/or field studies, including those aimed at developing new vaccines and for investigations into parasite genetics, population biology, and epidemiology.

For studies that define parasite numbers in tissue samples via a gDNA-based qPCR approach to be compared with each other, it is essential that a standardised protocol be adopted. A thorough understanding of the life cycle of the species under study is essential for effective interpretation of results and to avoid those stages of development/reproduction that may skew data. In this study we specifically sampled and homogenised whole caecal pairs to avoid the introduction of bias associated with uneven parasite replication and/or distribution within and between caeca. For *E. tenella* sampling at five dpi gave a balance between sensitivity (by targeting the massive numbers of parasites present within the developing third generation schizonts [53]), and reproducibility (by avoiding the developing macro- and microgametes [54]). Even small variations in the ratio of macro- to microgametocytes could skew the association between final numbers of oocysts and parasite genome numbers observed during gametogony [55,56]. Although definitive sex ratios remain unknown for any coccidian species, Reece et al. [57] found significant variation in the sex ratios of *Plasmodium chabaudi* gametocytes in infected MF1 mice that were parasite-adjusted in response to the presence of unrelated conspecifics. Although *Plasmodium* is not a coccidian, this phenomenon is likely to occur in other apicomplexans such as *Eimeria* so sampling during gametogony could add significant unexpected variation to the assay in the absence of a genetically homogeneous infection.

In developing this protocol we assessed amplification of a portion of each of four genetic markers within the chicken genome using previously validated assays to serve as reference sequences for the purposes of qPCR normalisation. Our final choice of target (152 nucleotides of the *actb* gene) was influenced by the specificity, repeatability (i.e., short-term precision; see [23]), and assay efficiency for each locus. Overall, there was minimal separation among *actb*, *β_2_m*, and *tbp* in as much as amplification of each locus resulted in highly comparable coefficients of determination (*R^2^*), standard curves, amplification factors, and assay efficiencies (see Table 2). The determining factor was the mean melting temperature of each locus, which suggested that the qPCR reaction amplifying *actb* was more specific compared to that for either *β_2_m* or *tbp*. In assessing efficiency, which can be influenced by amplicon length, G + C content and secondary structure, we endeavoured to obtain a standard curve with a slope of − 3.322, which would theoretically yield an amplification factor of 2.0, and an assay efficiency of 100% (i.e., DNA copies effectively doubling at each cycle of the PCR). Although variation in efficiency of between 90–115% is considered acceptable, efficiencies > 100% can indicate non-target fluorescence or DNA saturation, which results in reduced change in Cq scores at higher sample concentrations, causing a slope compression that inflates efficiency [48]. In the future, in order to bring efficiency closer to 100%, use of a target-specific probe would be preferred using TaqMan qPCR or a similar technology. Introducing this technology (i.e., multiplexed qPCR) would have the additional benefits of i) increased sample throughput, ii) reduced sample handling thereby decreasing opportunities for operator induced errors or those that result as a consequence of multiple freeze/thaw events, iii) reduced reagent/consumable cost, and iv) the need for large sample sizes, especially when samples are precious.

In conclusion, this study represents a breakthrough to quantifying *Eimeria* replication with particular relevance to experimental settings of infection. Quantitative real-time PCR is capable of detecting and measuring minute quantities of DNA from a variety of biological/environmental materials, which can be stored prior to in-depth evaluation or retrospective re-evaluation. Access to this medium-/high-throughput tool to measure *Eimeria* genome numbers provides a unique opportunity to investigate key aspects of *Eimeria* biology and control on a scale not previously accessible using FOC/lesion scoring. Importantly, the qPCR technique provides an unprecedented opportunity to i) reduce experimental animal group sizes without compromising statistical quality, a reduction in line with NC3Rs and ii) investigate the genetic basis of resistance/susceptibility to eimerian infection, providing a quantifiable phenotype amenable to quantitative trait locus mapping. In the future, comparative genetic studies of isolates with differing phenotypic traits linked to parasite–host interplay, virulence and pathogenicity as well as disease, together with host genetics, will be critical to understanding coccidiosis and improving anticoccidial control. Consequently, this study's immediate benefits are directly connected to the commercial poultry industry and associated management sectors, as well as academic research institutions.

## Conflict of interest

The authors have no conflict of interest.

## Figures and Tables

**Table 1:** Primer pairs employed to amplify chicken cytoplasmic beta-actin (*actb*), beta-2 microglobulin (*β_2_m*), glyceraldehyde 3-phosphate dehydrogenase (*gapdh*) and tata-binding protein (*tbp*) gene fragments, which were evaluated here as reference sequences for qPCR normalisation.

Target gene	Primer identity	Sequence (5′ to 3′)	Theoretical annealing temperature (°C)	Amplicon size (bp)	Primer references	GenBank accession nos. for locus	Primer location (from 5′ end)	Gene references
*actb*	Forward — actbF	GAGAAATTGTGCGTGACATCA	60	152	[58]	X00182	3003–3023	[41]
	Reverse — actbR	CCTGAACCTCTCATTGCCA	60				3136–3154	
*β_2_m*	Forward — b2mF	GCAAACCTCTGTCTTTCGGC	60	126	this study	Z48922	3191–3210	[42]
	Reverse — b2mR	ATGTTCAGACCAGAGCCTGC	60				3297–3316	
*gapdh*	Forward — GAPDH_For1	CGCAAGGGCTAGGACGG	60	98	[59]	M11213	220–236	[43]
	Reverse — GAPDH_Rev1	GCGCTCTTGCGGGTACC	60				301–317	
*tbp*	Forward — tbpF	TAGCCCGATGATGCCGTAT	62	147	[58]	NM_205103	144–162	[44]
	Reverse — tbpR	GTTCCCTGTGTCGCTTGC	60				273–290	

**Table 2:** Quantitative real-time PCR test results for a *Gallus gallus domesticus* gDNA dilution series over five orders of magnitude (10^4^–10^0^) for the amplification of cytoplasmic beta-actin (*actb*), beta-2 microglobulin (*β_2_m*), glyceraldehyde 3-phosphate dehydrogenase (*gapdh*), and tata-binding protein (*tbp*) protein-encoding gene fragments. Mean and standard deviation are provided for the coefficient of determination, slope, amplification factor and efficiency of three replicate reactions targeting the four loci.

				Coefficient of determination (*R*^2^)		Slope		Amplification factor		Efficiency (%)	
Locus	Repeat	Template	Linear range	Value	Mean ± SD	Value	Mean ± SD	Value	Mean ± SD	Value	Mean ± SD
*actb*	1	Genomic DNA	1.0^5^–1.0^0^	0.990	0.985 ± 0.009	− 2.920	− 3.048 ± 0.198	2.253	2.124 ± 0.119	120.022	113.452 ± 9.992
	2	Genomic DNA	1.0^4^–1.0^0^	0.990		− 3.276		2.020		101.953	
	3	Genomic DNA	1.0^4^–1.0^0^	0.975		− 2.948		2.100		118.380	
*β_2_m*	1	Genomic DNA	1.0^5^–1.0^0^	0.974	0.983 ± 0.009	− 2.938	− 3.066 ± 0.270	2.185	2.121 ± 0.123	118.961	112.984 ± 13.256
	2	Genomic DNA	1.0^4^–1.0^0^	0.984		− 3.376		1.979		97.792	
	3	Genomic DNA	1.0^4^–1.0^0^	0.992		− 2.884		2.200		122.198	
*gapdh*	1	Genomic DNA	1.0^5^–1.0^0^	0.978	0.975 ± 0.009	− 1.910	− 2.400 ± 0.427	3.300	2.685 ± 0.533	233.857	170.529 ± 54.955
	2	Genomic DNA	1.0^4^–1.0^0^	0.965		− 2.690		2.354		135.368	
	3	Genomic DNA	1.0^4^–1.0^0^	0.983		− 2.601		2.400		142.364	
*tbp*	1	Genomic DNA	1.0^5^–1.0^0^	0.992	0.988 ± 0.006	− 2.867	− 3.053 ± 0.205	2.247	2.137 ± 0.114	123.253	113.247 ± 10.632
	2	Genomic DNA	1.0^4^–1.0^0^	0.981		− 3.019		2.144		114.405	
	3	Genomic DNA	1.0^4^–1.0^0^	0.992		− 3.273		2.020		102.083	

**Table 3:** *Eimeria tenella* replication and impact defined by average faecal oocyst count (FOC) per bird, lesion score and qPCR quantified intracellular genomes.

Group	Dose (oocysts per bird)	FOC (Log_10_ oocysts/bird)	Lesion score		Log_10_ parasite genomes/mg host tissue	
			5 dpi	8 dpi	5 dpi	8 dpi
1	Uninfected	ND	nd	0^a^	nd	0
2	500	6.67 ± 0.73	1.3 ± 0.5^a^	1.2 ± 0.7^a^	5.2 ± 0.1^a^	4.9 ± 0.2
3	1500	7.14 ± 0.69	2.0 ± 0.6^a^	2.8 ± 0.4^b^	5.6 ± 0.2^b^	4.6 ± 0.4
4	4500	7.58 ± 0.37	3.6 ± 0.5^b^	3.0 ± 0.3^b^	6.1 ± 0.2^c^	5.0 ± 0.4
	*R*^2^	0.9969	0.7384	0.6302	0.9942	0.0059

ND = none detected; the limit of detection was 4.3 Log_10_ oocysts/bird. nd = not done. Statistically significant differences in columns indicated by different superscript letters.
